# Prevalence of *Babesia microti* Co-Infection with Other Tick-Borne Pathogens in Pennsylvania

**DOI:** 10.3390/microorganisms12112220

**Published:** 2024-11-01

**Authors:** Lovepreet S. Nijjar, Sarah Schwartz, Destiny Sample Koon Koon, Samantha M. Marin, Mollie E. Jimenez, Trevor Williams, Nicole Chinnici

**Affiliations:** Dr. Jane Huffman Wildlife Genetics Institute, East Stroudsburg University, East Stroudsburg, PA 18301, USA; lnijjar@esu.edu (L.S.N.); sschwart10@esu.edu (S.S.); dsample@esu.edu (D.S.K.K.); smarin1@esu.edu (S.M.M.); mjimenez8@esu.edu (M.E.J.); trevorw4444@gmail.com (T.W.)

**Keywords:** *Babesia microti*, *Borrelia burgdorferi*, *Anaplasma phagocytophilum*, co-infection, tick-borne pathogens, *Ixodes* ticks, protozoan infections, vector-borne diseases, disease management

## Abstract

*Babesia microti* is a protozoan that infects red blood cells, causing hemolytic anemia and flu-like symptoms in humans. Understanding co-infections is crucial for the better diagnosis, treatment, and management of tick-borne diseases. This study examined the prevalence of *Babesia microti* co-infection with other prevalent tick-borne pathogens in Pennsylvania. The dataset acquired from the Dr. Jane Huffman Wildlife Genetics Institute included passive surveillance data from *Ixodes* spp. from 2021 to 2023. Submitted ticks were screened for tick-borne pathogens using species-specific TaqMan qPCR. Of the 793 *B. microti*-positive ticks pulled for analysis, 65.0% were co-infected with other pathogens (n = 516). Notably, 60.9% of the *B. microti*-positive ticks were co-infected with *Borrelia burgdorferi*, 10.2% with *Anaplasma phagocytophilum* Ap-ha, and 7.5% carried a triple co-infection with *B. burgdorferi* and *A. phagocytophilum* Ap-ha. The rates of *B. microti* infection and its co-infections are on the rise, with patterns observed in Pennsylvania and other regions of the USA. While other studies have collected both nymphal and adult ticks to screen for co-infections in Pennsylvania, our study stood out as a unique contribution to the field by focusing exclusively on *B. microti*-positive ticks. The continued monitoring of tick-borne co-infections is vital to prevent misdiagnosis and ensure effective treatment regimens.

## 1. Introduction

*Babesia microti*, a hemoprotozoan of the genus *Babesia*, is primarily transmitted by the *Ixodes scapularis* tick, also known as the blacklegged tick [1]. Transmission occurs when an infected tick bites a host, injecting the parasite into the bloodstream. *B. microti* evades red blood cells (RBCs), where it begins multiplying, leading to their destruction and causing hemolytic anemia [1,2]. Effective transmission from tick to host occurs in an estimated period of from 36 to 72 h, and if the tick is removed before this period, the risk of transmission is notably reduced. Babesiosis presents with symptoms such as headache, fatigue, chills, fever, sweats, and nausea, which can resemble flu-like conditions. Severe cases may lead to complications like multi-organ dysfunction, including respiratory distress, congestive heart failure, renal failure, splenic rupture, disseminated intravascular coagulation (DIC), hepatitis, or coma [3]. Individuals who are immunocompromised, have had their spleens removed, or have preexisting liver or kidney conditions are at higher risk for severe symptoms [3]. Some infected individuals may remain asymptomatic, complicating diagnosis and disease management [1]. Babesiosis remains non-mandatorily reportable in Pennsylvania despite a 20-fold case increase over 12 years reported by the Pennsylvania Department of Health in 2018 [4]. 

In Pennsylvania, the blacklegged tick is associated with several other human pathogens, including *Borrelia burgdorferi*, *Anaplasma phagocytophilum*, *Borrelia miyamotoi*, and Powassan virus [5,6,7,8,9]. The causative agent of Lyme disease, *B. burgdorferi* sensu lato (s.l), is a spirochetal bacterium. The Center for Disease Control and Prevention (CDC) reported a total of 62,551 Lyme disease cases in 2022 following a revised case definition [10]. Lyme disease is most notably diagnosed by the erythema migrans (EM) rash; however, in the absence of the rash, symptoms can be non-specific, resulting in misdiagnosis and delayed treatment. Untreated Lyme disease cases have resulted in late dermatologic, cardiac, neurologic, and joint manifestations. Additionally, research has shown that a subset of patients, following appropriate antibiotic treatment, continue to have persistent symptoms, impacting their quality of life [11]. The early removal of ticks can prevent Lyme disease, as *B. burgdorferi* requires 24 to 72 h to transmit, while Powassan virus can spread in just 15 min [9,12,13]. Powassan virus is a Flavivirus with two distinct genetic lineages: lineage I and deer tick virus (lineage II). In North America, while rare, Powassan virus is the only Flavivirus that may cause cases of encephalitis, meningoencephalitis, aseptic meningitis, or non-neuroinvasive infections [14]. The rapid transmission from tick to host is attributed to the virus’s affinity for the salivary glands. A total of 46 neuroinvasive cases of Powassan virus have been reported to the CDC in 2024, including a single case in Pennsylvania [15]. Hard tick relapsing fever, caused by *B. miyamotoi*, is a sister bacterium to *B. burgdorferi* causing non-specific febrile illness. However, unlike Lyme disease, cases of hard tick relapsing fever do not constitute a nationally notifiable disease, and thus, the overall impact on human health is less understood [16]. *B. miyamotoi* and deer tick virus can undergo vertical transmission while other pathogens rely on blood meals, making nymphs and adults the primary vectors [9,17]. The emergence of new pathogens and variants in blacklegged ticks raises public health concerns. *A. phagocytophilum*, a rickettsial bacterium, is the second-most-reported tick-borne disease, with 6729 cases in 2021 [18]. There are two variants of *A. phagocytophilum*: one affecting humans (Ap-ha) and another linked to ruminants (Ap-v1) [9,19]. Differentiating these strains is essential for effective tick surveillance as blacklegged ticks can harbor multiple pathogens, leading to co-infections [9]. Understanding co-infection prevalence, transmission dynamics, risk factors, and clinical manifestations is essential for effective prevention and treatment.

The study of co-infections is crucial due to their complex impact on pathogen interactions and disease outcomes [20,21]. A co-infection is defined as an instance where a tick is harboring two or more tick-borne pathogens. When multiple pathogens simultaneously infect a host, the resulting interactions can alter pathogen behavior and potentially increase disease severity or complicate treatment. Pathogen interactions within a vector and host can influence replication and transmission, impacting overall disease progression [20]. Co-infections can also affect the immune system, leading to immune suppression or dysregulation, which may make the host more susceptible to other infections and complicate health management [20,22,23]. Accurate disease surveillance and epidemiology depend on understanding these interactions, which help in identifying high-risk populations and tailored public health interventions. Co-infections can complicate diagnosis and treatment, necessitating targeted treatment plans to prevent ineffective treatments and improve patient care. Additionally, co-infections can influence resistance patterns, potentially leading to the development of antibiotic or antiviral resistance [24]. Studying co-infections contributes to a deeper understanding of disease mechanisms and pathogenesis, which is essential for developing new therapeutic strategies and enhancing disease prevention [20,21].

Investigating *B. microti* co-infections is a priority due to the public health concern as disease progression is complicated by non-specific symptoms that can lead to misdiagnosis and medical errors in treatment protocols. Additionally, babesiosis is a tick-borne pathogen that can be transmitted through blood transfusions [25,26]. Alone, *B. microti* has a low ecological fitness due to poor transstadial transmission and poor reservoir transmission, creating the risk of emergence models that do not agree with the actual spread and emergence of babesiosis [27]. Meanwhile, more recent studies have highlighted a synergistic relationship between *B. microti* and *B. burgdorferi*, suggesting that *B. burgdorferi* can promote pathogen maintenance and the geographical establishment of *B. microti* [28]. In this study, we focused specifically on *B. microti* co-infections due to the proposed synergistic relationship as co-infections with Lyme disease and babesiosis pose a significant public health threat. The severity of babesiosis is influenced by factors such as underlying health conditions, the species involved, and co-infections with other tick-borne pathogens [25]. In regions where tick-borne pathogens are endemic, where co-infections are common, research on improved diagnostics, treatment strategies, and the dynamics of co-infections is critical. Understanding the complexities of *B. microti*-positive ticks with direct associations with humans through passive surveillance data can lead to the better management of the disease and reduce its impact on public health, making it a priority to become reportable in the state of Pennsylvania. 

## 2. Materials and Methods

### 2.1. Tick Selection

**Figure 1:**
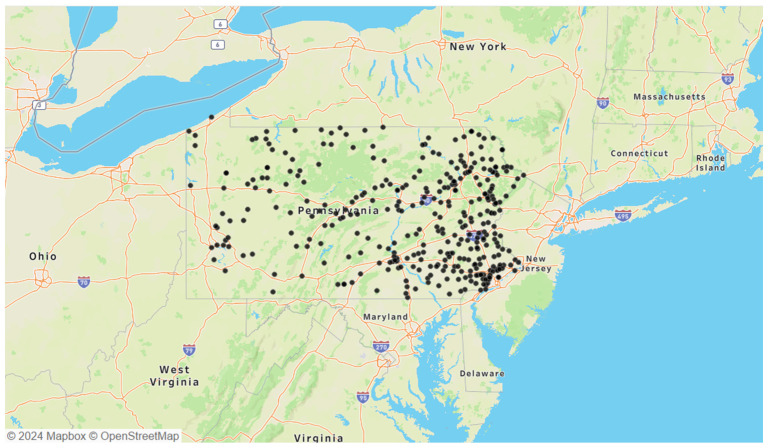
Locations of all 793 *B. microti*-positive ticks obtained from passive surveillance data collected between 2021 and 2023.

The selected *B. microti*-positive ticks were *Ixodes* species, tested through a passive surveillance program conducted by the Dr. Jane Huffman Wildlife Genetics Institute in Pennsylvania from 2021 to 2023 (Figure 1). Due to the nature of passive surveillance efforts, variations in total *B. microti*-positive ticks were observed between years: 2021 had 221 ticks, 2022 had 216 ticks, and 2023 had 356 ticks. Additionally, the geographic distribution of *B. microti*-positive ticks in Pennsylvania showed a concentration in the eastern portion of the state. Twenty-eight samples from 2021 were excluded due to the absence of the Single-Nucleotide Polymorphism (SNP) genotyping assay, which was developed later that year to differentiate between the two *A. phagocytophilum* strains.

### 2.2. Pathogen Detection

*Ixodes* ticks were extracted with midsagittal cuts with a disposable scalpel followed by total nucleic acid extraction via magnetic glass particle technology on the MagNA Pure 96 System (Roche Diagnostics, Indianapolis, IN, USA) and QIAamp Viral RNA kits (Qiagen, Redwood City, CA, USA), following the manufacturer’s protocols. To prevent contamination, small extraction groups (of less than 31) with an extraction blank were employed. The extraction blank controls, which included all reagents and excluded tick samples, were screened for potential extraction contaminants. All 793 ticks underwent qPCR testing for *B. microti*, *B. burgdorferi*, *A. phagocytophilum*, *B. miyamotoi*, Powassan virus lineage I/II, and an internal positive tick control for Ixodidae to confirm successful extraction. Validated TaqMan^TM^ multiplex assays were used for species-specific pathogen detection and a SYBR green reverse transcriptase assay with melt curve analysis for Powassan virus lineage I/II (Table 1) [9]. Samples testing positive for *A. phagocytophilum* were further analyzed using an SNP Genotyping assay and evaluated with an allelic discrimination plot [9,29]. Additionally, samples that were positive for Powassan virus lineage I/II were screened on a lineage-specific TaqMan^TM^ assay to differentiate deer tick virus and lineage-I Powassan virus. Amplification was performed using a StepOnePlus Real-Time PCR System (Applied Biosystems, Waltham, MA, USA) and an Applied Biosystems QuantStudio5 (Thermo Fisher Scientific, Waltham, MA, USA) instrument, following the recommended cycling conditions for TaqMan^TM^ Fast Advanced Master Mix (Applied Biosystems, Waltham, MA, USA) for qPCR and adjusted primer concentrations. A sample was considered positive only if it met predefined amplification thresholds and C*q* values of 40 cycles or less. Each assay included both positive and negative controls, with nuclease-free water substituting DNA in the negative control. Known synthetic positives for *B. microti*, *B. burgdorferi*, *A. phagocytophilum*, *B. miyamotoi*, and Powassan virus lineage I/II were used as the positive controls and purchased from GeneWiz (South Plainfield, NJ, USA). All assays were validated using standards and guidelines developed internally by the Dr. Jane Huffman Wildlife Genetics Institute, consistent with CDC guidelines [9,30].

### 2.3. Exploratory Data Analysis

Exploratory data analysis (EDA) was conducted using Tableau (2023.3) and R statistical software (version 4.4.1). The code utilized in this research can be found in the Supplementary Materials. Tableau primarily served as a data visualization tool for geospatial mapping and creating plots. In R, the data were converted into a logical format to create ecological objects defined by individual pathogens, a necessary step for generating association rules using the arules() package in R [36]. The apriori algorithm was employed to uncover relationships between various pathogens, with parameters for support and confidence set to ensure meaningful associations [36]. The focus was on association rules involving *B. microti* to explore its co-infection patterns. The rules were filtered to include instances where *B. microti* appeared on the right side of the rule, allowing for the identification of notable associations and co-infection patterns relevant to the study. The rules were then examined and sorted by support, which indicated the frequency of these associations. A support threshold of 0.01 was applied to capture co-infections appearing in at least 1% of ecological interactions. By requiring a confidence of 1, only perfectly consistent rules, where the relationship held 100% of the time, were generated. A bar plot was created using ggplot2 to display the support of each rule involving *B. microti* [37]. In analyzing a dataset exclusively comprising *B. microti*-positive ticks, EDA proved to be the most effective approach for uncovering patterns in co-infection data. EDA portrayed key ecological trends and anomalies while concentrating on the relationships between *B. microti* and other pathogens.

## 3. Results

**Figure 2:**
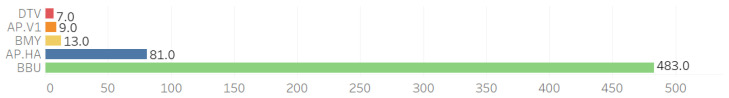
Individual counts of other pathogens observed with the 793 *B. microti*-positive ticks.

Co-infections were observed across five pathogens alongside *B. microti* (BMI): *B. burgdorferi* (BBU), *B. miyamotoi* (BMY), deer tick virus (DTV), *A. phagocytophilum* Ap.ha (Ap-ha), and *A. phagocytophilum* Variant 1 (Ap-v1) (see Figure 2).

**Figure 3:**
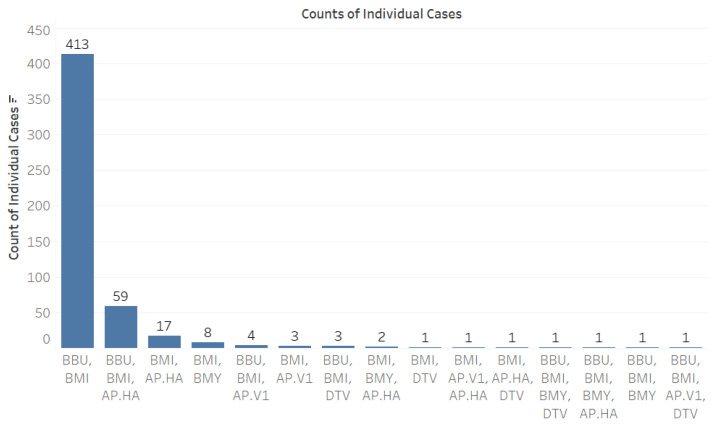
Counts of individual cases of co-infection among tick-borne pathogens. Abbreviations for pathogens: *B. microti* (BMI), *B. burgdorferi* (BBU), *B. miyamotoi* (BMY), deer tick virus (DTV), *A. phagocytophilum* Human-Active (Ap-ha), and *A. phagocytophilum* Variant 1 (Ap-v1).

**Figure 4:**
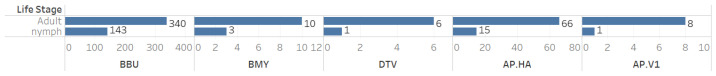
Counts of individual cases of co-infection among tick-borne pathogens by tick life stage.

When analyzing co-infection combinations involving the 793 *B. microti*-positive ticks, we observed a notable prevalence of co-infection with *B. burgdorferi*, with 413 positive results recorded (Figure 3). Additionally, there were fifty-nine instances of triple co-infection involving *B. burgdorferi* and Ap-ha, seventeen cases with Ap-ha, and eight cases with *B. miyamotoi* (Figure 3). The highest rates of co-infection were found in adult *Ixodes* ticks (Figure 4).

**Figure 5:**
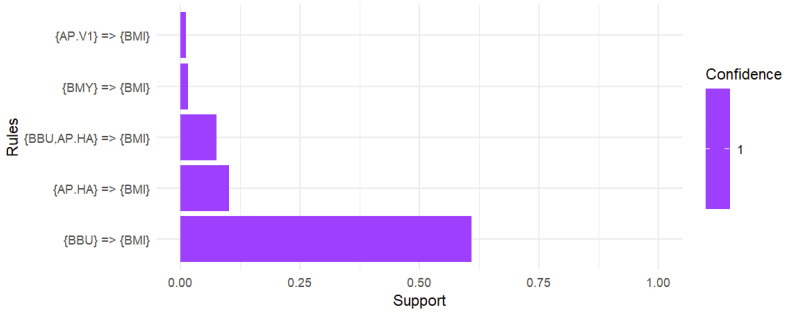
Association rules analysis of the co-infection with *B. microti.* A support threshold of 0.01 captured co-infections in at least 1.0% of interactions while a confidence of 1 produced strong associations with *B. microti.* A total of 60.9% of ticks positive for *B. microti* were co-infected with *B. burgdorferi.* The likelihood of co-infection with Ap-ha was 10.2% while the chance of harboring a triple infection with *B. burgdorferi* and Ap-ha alongside *B. microti* was 7.5%. Additionally, the presence of *B. microti* suggested a 1.6% chance of co-infection with *B. miyamotoi* and a 1.1% chance of co-infection with Ap-v1.

In the associationrules mining analysis conducted on a sample of 793 *B. microti*-positive ticks, including both adult and nymphal ticks, the data revealed that 60.9% of the ticks exhibited co-infection with *B. burgdorferi* while 10.2% showed co-infection with Ap-ha. Notably, 7.5% of the ticks were identified with a triple co-infection involving both *B. burgdorferi* and Ap-ha. Additionally, 1.6% of the ticks were co-infected with *B. miyamotoi* and 1.1% were identified with Ap-v1. It is also worth noting that deer tick virus was eliminated from the analysis due to not meeting the support threshold of 0.01 (Figure 5).

## 4. Discussion

This study focused on *B. microti* co-infections with other tick-borne pathogens in Pennsylvania, using passive surveillance data from the Dr. Jane Huffman Wildlife Genetics Institute (2021–2023). The ecological patterns identified in our exploratory data analysis were consistently reflected across the dataset, highlighting their relevance. Given the unique nature of our study, which focused exclusively on *B. microti*-positive ticks, we prioritized EDA over traditional statistical methods to better capture these insights. Among 793 *B. microti*-positive ticks, 65.0% (n = 516) were co-infected with other pathogens. If a tick was positive for *B. microti*, there was a 60.9% chance it was also infected with *B. burgdorferi*. Additionally, the likelihood of co-infection with Ap-ha was 10.2%. Importantly, if the tick carried *B. microti*, there was a 7.5% chance it also harbored both *B. burgdorferi* andAp-ha. Furthermore, a positive result for *B. microti* suggested a 1.6% chance of co-infection with *B. miyamotoi* and a 1.1% chance of having Ap-v1. Our study also demonstrated that adult ticks exhibited a higher prevalence of pathogens than nymphs, indicating a greater occurrence of co-infections in adult ticks. Analyzing both adult and nymph life stages is essential in tick surveillance. Adult ticks, having completed two blood meals, are exposed to more reservoirs, which can assist with understanding and detecting rare pathogens. Meanwhile, nymphs are a greater public health concern due to their small size and difficulty for human detection.

High co-infection rates can be influenced by environmental interactions, which include pathogen–host, pathogen–vector, vector–host–pathogen, and pathogen–pathogen interactions [38]. Pathogen–host interactions contribute to reservoir host abundance. Small mammals, such as white-footed mice and shrews, serve as a reservoir for tick-borne pathogens and have the ability to maintain more than one pathogen. This suggests dual pathogen acquisition from a single blood meal due to horizontal transmission dynamics [5,9]. Furthermore, pathogen–host interactions can influence the transmission of pathogens within the reservoir host to offsprings, referred to as vertical transmission. Evidence of vertical transmission in reservoirs has been documented for *B. microti*; however, it is less understood for *B. miyamotoi* and *A. phagocytophilum* [38]. Our findings suggest that the high chance of co-infection with Ap-ha compared to Ap-v1 may indicate a reservoir prevalence, influenced by the host preferences of the two variants. As Ap-v1 is seen in cervids as opposed to small mammals, future studies should investigate the potential facilitation between Ap-ha anaplasmosis and babesiosis in mouse models. The complexity of the pathogen–vector interaction includes maintaining a pathogen transstadial and undergoing vertical transmission, another source for the co-occurrence of pathogens within a single vector. This phenomenon is where ticks acquire pathogens through vertical transmission from adult to larvae; however, this is only observed for *B. miyamotoi* and Powassan virus [9]. In our findings, *B. miyamotoi* was the only vertical transmitted pathogen that filtered through the association rules analysis at a 1.6% chance of being co-infected with *B. microti*. This could contribute to lower overall rates of *B. miyamotoi* infection or an implication that transovarian transmission is not enough to establish high occurrence when compared to horizontal transmission. Additionally, pathogen–vector–host interactions can be described by horizontal transmission through co-feeding, where vectors acquire pathogens from each other due to close proximity feeding, contributing another cause to these co-infection patterns. *B. burgdorferi* and *B. microti* co-infection rates are higher than expected when assessed independently, suggesting pathogen–vector–host interactions for both pathogens, facilitating vector acquisition during a single blood meal [5,8,9]. Most notably, the pathogen–pathogen interaction may best describe the higher co-infection rates, involving *B. burgdorferi* and *B. microti*, as well as *A. phagocytophilum* with both pathogens, observed within this study. Independently, *B. microti* prevalence is low, with a range of prevalence rates from 2.5% to 5.5% in Pennsylvania [9]. The positive interaction or facilitation between *B. microti* and *B. burgdorferi* dynamics agrees with the high co-occurrence of 60.9% in our study, further supporting the pathogen–pathogen synergistic relationship [27]. Additionally, the further monitoring of lower prevalence pathogens is necessary to provide insights, such as is the case with *B. miyamotoi*, which persists at approximately 1.0% across the northeastern, Midwest, and mid-Atlantic United States [9,39]. Despite its low abundance, *B. miyamotoi* was supported by the association rules analysis, suggesting a 1.6% chance of co-infection with *B. microti*. This may represent the first indication of a positive interaction between both *B. miyamotoi* and *B. microti* as previous tick studies had not found significant differences between expected and observed rates [9,39]. As the prevalence of *B. miyamotoi* continues to be monitored by surveillance programs, it may provide new insights into the facilitation of co-infections. Diseases that are not nationally notifiable, such as that caused by *B. miyamotoi*, or those not required to be reported at the state level, such as that caused by *B. microti*, rely on tick surveillance data to provide spatial and temporal exposure estimates. These findings highlight the importance of a comprehensive surveillance program that screens for multiple pathogens, serving as a critical tool for early diagnosis and public health intervention, similar to the efforts at the Dr. Jane Huffman Wildlife Genetics Institute. The high rates of co-infection seen in this study, particularly involving *B. microti* and *B. burgdorferi*, mirror trends observed in other regions of the USA., emphasizing the increasing complexity of managing tick-borne diseases [40]. This underscores the importance of addressing co-infections as patients infected with multiple pathogens may exhibit more severe symptoms and respond inadequately to standard treatments [40]. The rising prevalence of *B. microti* and its co-infections calls for enhanced surveillance and diagnostic strategies, particularly in Pennsylvania, where these co-infections are becoming more prominent [8,9]. Continued monitoring is crucial to improving treatment protocols and reducing public health risks associated with these pathogens.

While positive interactions were observed for *B. microti* co-infections with *B. burgdorferi*, *A. phagocytophilum,* and *B. miyamotoi*, there was lack of support through the association rule analyses between deer tick virus and *B. microti*. The lack of support may indicate a negative interaction or competition between deer tick virus and *B. microti*. However, this may have been influenced by the overall low abundance of deer tick virus independently. The CDC reports a total of sixteen confirmed human cases from 2004 to 2023 in Pennsylvania, all of which were neuroinvasive [41]. The literature on deer tick virus co-infections in human cases is limited to non-existent. However, an active tick surveillance program in Pennsylvania agreed with the lack of significant co-infections between deer tick virus and *B. microti* based on the analysis of expected and observed prevalence [9]. The continued surveillance, both active and passive, of deer tick virus is necessary to further investigate the interactions between these tick-borne pathogens.

While other studies have collected both nymphal and adult ticks to screen for co-infections in Pennsylvania, our study stood out as a unique contribution to the field by focusing exclusively on *B. microti*-positive ticks [6,8,9]. By examining co-infection dynamics within this defined group, we enhanced our understanding of the ecological and epidemiological implications of *B. microti* in the context of tick-borne diseases. Despite its poor ecological fitness, *B. microti* has emerged in Lyme-disease-endemic areas. Studies have shown that co-infection with *B. burgdorferi* and *B. microti* is common, promoting the transmission and emergence of *B. microti* in the enzootic cycle [27]. Additionally, the OspA vaccine, originally developed to target *B. burgdorferi*, not only reduces co-infections in ticks but also lowers *B. microti* prevalence in the reservoir host, indirectly reducing babesiosis risk [42]. This result highlights the interconnected nature of these pathogens and the need to target both to mitigate their combined impact. This study revealed that 65.0% of *B. microti*-positive ticks were co-infected with other pathogens, highlighting critical implications for tick-borne disease surveillance. Individuals co-infected with these pathogens tend to experience heightened symptoms and worse clinical outcomes compared to those with a singular infection, complicating diagnosis and potentially delaying appropriate treatment [27]. Patients with babesiosis co-infections require combination therapies as single-agent treatments are often inadequate in managing these co-infections. Studies have shown that babesiosis, particularly when paired with other infections like Lyme disease or anaplasmosis, can lead to greater systemic issues including respiratory failure and renal complications. While human co-infection with *B. miyamotoi* and *B. microti* has been documented, the potential complications and severity of disease remain unclear [43]. These findings suggest that co-infection worsens disease outcomes, requiring careful monitoring and potentially aggressive treatments such as combined antimicrobial therapies [44]. For healthcare providers, understanding these risks reinforces the importance of early diagnosis and tailored treatment protocols for patients with tick-borne co-infections.

The geographic distribution of the observed *B. microti*-positive ticks suggests a potential western emergence in Pennsylvania as it establishes within reservoir and vector populations. The significant facilitation of these co-infections via blood meals has been reported in *I. scapularis* larvae [5,27]. Thus, the density of *B. microti*-positive ticks in the eastern region likely reflects environmental factors and host availability, raising concerns about co-infection interactions [9]. However, selection bias inherent in passively acquired data limits the detection of all cases. While active surveillance is more comprehensive, it requires extensive resources, making passive surveillance more practical despite its limitations [45,46]. Our passive surveillance data, relying on tick-submissions acquired from human and animal hosts, may contain errors due to their self-reporting nature, affecting the accurate reports of *B. microti* spatial distribution. However, the nature of this self-reporting also brings insight on incidental host exposures, including of humans and domesticated pets. Thus, the co-infections reported in this study offer direct insights into *B. microti* co-infections and can be used as helpful tools for public health awareness. The continuous monitoring of positive ticks is crucial to identify trends in pathogen emergence and disease risk.

In the years 2021–2023, there were 27,984 reported Lyme cases, 420 reported babesiosis cases, and 2556 reported anaplasmosis cases in Pennsylvania [47]. Babesiosis is not a mandatory reportable disease in Pennsylvania; however, the Pennsylvania Department of Health does receive reports of cases from healthcare providers that elect to do so [4]. The absence of mandatory reporting for babesiosis undermines efforts to effectively address this public health issue. This issue is particularly concerning, especially considering that in Connecticut, around 20% of patients admitted to the Yale New Haven Hospital for over a decade for severe babesiosis experienced cardiac complications. Most notably, three out of four patients who died had cardiac issues that factored into their fatalities [48]. Although Lyme disease cases are highly observed, there is limited published information on tick-borne disease co-infections in humans, particularly regarding whether trends observed in tick populations translate to human disease. Additionally, there was a case of a 70-year-old individual, exposed in the northeastern USA, who was diagnosed with Lyme disease, babesiosis, and anaplasmosis after presenting with fever and laboratory abnormalities following a suspected insect bite. While a co-infection with Lyme disease is relatively common, the simultaneous presence of all three pathogens is rare [49]. Our data revealed a notable 7.5% chance of a triple co-infection when *B. microti* was present. In Lyme-disease-endemic regions, medical practitioners should consider screening for co-infections including those of *B. microti* and *A. phagocytophilum* [27,40]. Furthermore, co-infections should be considered when patients present with prolonged flu-like symptoms that are not responding to recommended Lyme disease treatments [50]. The impact of these co-infections on the general population remains poorly understood, reinforcing the urgent need for further surveillance and research to assess their implications for public health.

To enhance the analysis of diagnostic challenges associated with co-infections in clinical settings, it is crucial to acknowledge the limitations of existing diagnostic tools. Currently, diagnostic testing is limited to single-pathogen resolution with a focus solely on Lyme disease. This diagnostic approach fails to identify the presence of multiple infections at once, which can lead to misdiagnosis or delayed treatment. Improved *B. miyamotoi* detection in human cases, despite the cross-reactivity seen in *B. burgdorferi* serological assays, needs to be of direct concern to properly investigate co-infection implications [51]. The increasing prevalence of co-infections, particularly in endemic regions, highlights the urgent need for improved diagnostic strategies to accurately identify multiple pathogens, thereby enhancing patient outcomes and public health surveillance.

## 5. Conclusions

Through the unique lens of association rules analysis and a focus on *B. microti*-positive ticks, this study identified advantageous interactions between the co-infections of tick-borne pathogens. Increased proportions of *B. microti* and co-infections with other pathogens highlight a shift in the landscape of vector-borne diseases. Notably, co-infection with Lyme disease and babesiosis shows a consistent pattern across various regions in the USA, including Pennsylvania [9,47]. This pattern is supported by numerous studies demonstrating the widespread nature of this co-infection. Less notably, this study found associations between babesiosis and less prevalent diseases, such as hard tick relapsing fever, and less-studied co-infection patterns, such as Ap-ha anaplasmosis. The continued surveillance of co-infections will enhance our understanding of advantageous versus competitive interactions between pathogens. Understanding the impact of co-infections on the immune system is crucial for developing effective treatment protocols [20,23,24]. Co-infections can complicate immune responses, potentially altering disease progression and treatment outcomes. Therefore, investigating the immunological effects of these co-infections is essential for refining treatment strategies and improving patient care [20,21]. The continued use of passive surveillance data can improve our understanding of the enzootic cycle through ticks directly associated with humans, ultimately aiding in developing targeted and effective interventions.

## Figures and Tables

**Table 1:** Primers and TaqMan^TM^ probe sequences used in this study.

Species	Gene	Primer Sequence	Probe Sequence	Source
*Babesia microti *	18s rRNA	F—CAGGGAGGTAGTGACAAGAAATAACA R—GGTTTAGATTCCCATCATTCCAAT	VIC—TACAGGGCTTAAAGTCT—MGBNFQ	[31]
*Borrelia burgdorferi *	16s-23S	F—GCTGTAAACGATGCACACTTGGT R—GGCGGCACACTTAACACGTTAG	6FAM—TTCGGTACTAACTTTTAGTTAA—MGBNFQ	[32]
*Borrelia miyamotoi *	16s-23S	F—GCTGTAAACGATGCACACTTGGT R—GGCGGCACACTTAACACGTTAG	ABY—CGGTACTAACCTTTCGATTA—QSY	[32]
*Anaplasma phagocytophilum *	Msp2	F—ATGGAAGGTAGTGTTGGTTATGGTATT R—TTGGTCTTGAAGCGCTCGTA	ABY—TGGTGCCAGGGTTGAGCTTGAGATTG—QSY	[33]
*Anaplasma phagocytophilum * variants	16s rRNA	F—ACATGCAAGTCGAACGGATTATTCT R—GCTATCCCATACTACTAGGTAGATTCCT	(Ap-ha) VIC—CTGCCACTAACTATTCT—MGB (Ap-v1) FAM—CTGCCACTAATTATTCT—MGB	[29]
* Powassan virus lineage I and II	NS5	F—CATAGCAAAGGTGAGATCCAA R-TCGCTGAGCTCCATTTATT		[34]
* Powassan lineage I	NS5	F—CATAGCGAAGGTGAGGTCCAA R-TTGCCGAGCTCCACTTGTT	QSY-CGCTTGGTCGGATGAACA-6FAM	[34]
Deer tick virus (Powassan lineage II)	NS5	F—GATCATGAGAGCGGTGAGTGACT R—GGATCTCACCTTTGCTATGAATTCA	BHQ-TGAGCACCTTCACAGCCGAGCCAG-6FAM	[34]
Ixodidae positive control	16s rRNA	F—AATACTCTAGGGATAACAGCGTAATAATTTT R—CGGTCTGAACTCAGATCAAGTAGGA	6FAM—AATAGTTTGCGACCTCGATGTTGGATTAGGAT-QSY	[35]

* Primers were modified to accommodate both lineages (Powassan lineage I and II) and to increase specificity for lineage discrimination (Powassan lineage I) [32].

## Data Availability

The processed data and R code presented in the study are included in the Supplementary Materials; further inquiries can be directed to the corresponding authors.

## Supplementary Materials

The following supporting information can be downloaded at: https://www.mdpi.com/article/10.3390/microorganisms12112220/s1, Figure S1: Co-infections in 2021 with *B. microti*; Figure S2: Co-infections in 2022 with *B. microti*; Figure S3: Co-infections in 2023 with *B. microti*.
