# Supplementary material for: Prevalence of Babesia microti Co-Infection with Other Tick-Borne Pathogens in Pennsylvania
Source: Microorganisms. 2024 Nov 1;12(11):2220. doi: 10.3390/microorganisms12112220 (PMC11596951; doi:10.3390/microorganisms12112220)
Supplement: Supplementary file 1 [file microorganisms-12-02220-s001.zip › Supplementary Figures.pdf]

Supplementary Materials

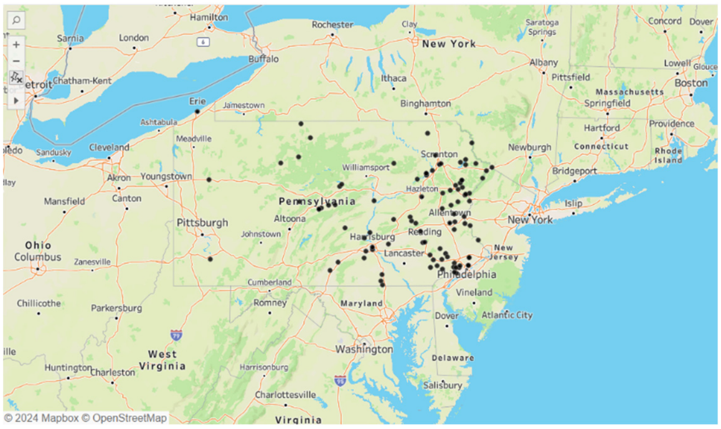

Figure S1. Co-infections in 2021 with *B. microti*

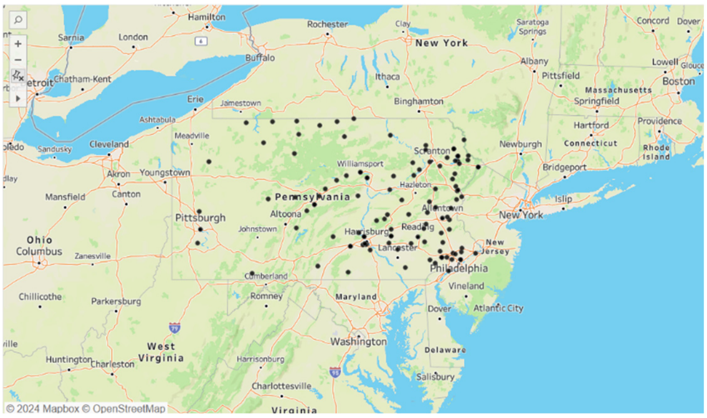

Figure S2. Co-infections in 2022 with *B. microti*

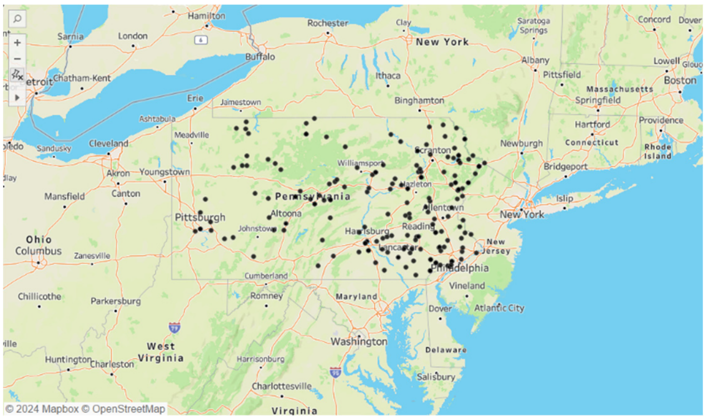

Figure S3. Co-infections in 2023 with *B. microti*

*Due to the nature of passive surveillance efforts, variations in total *B. microti*-positive ticks were observed between years: 2021 had 221 ticks, 2022 had 216 ticks, and 2023 had 356 ticks.
